# Study of Mechanics and Durability of Non-Spontaneous Combustion Coal Gangue Coarse-Aggregate High-Performance Concrete

**DOI:** 10.3390/ma17071534

**Published:** 2024-03-27

**Authors:** Zhigang Wang, Hongqiang Ma, Xiaoyan Niu

**Affiliations:** 1 Architectural Engineering Institute, Guangdong University of Petrochemical Technology, Maoming 525000, China; alexsea@gdupt.edu.cn; 2 College of Civil Engineering and Architecture, Hebei University, Baoding 071002, China; 3 Engineering Research Center of Zero-Carbon Energy Buildings and Measurement Techniques, Ministry of Education, Hebei University, Baoding 071002, China

**Keywords:** non-spontaneous combustion coal gangue, high-performance concrete, durability, GM(1,1) prediction model, life prediction

## Abstract

The coal gangue coarse-aggregate content in ordinary concrete should not be too large. In order to further improve the utilization rate of coal gangue coarse aggregate, this study used the principle of “strong wrapped weak” to prepare high-performance concrete. This study considered four factors, namely, water–binder (W/B) ratios, non-spontaneous combustion coal gangue (NCCG) coarse-aggregate contents, fly ash–slag mass ratios, and silica fume coating to prepare high-performance concrete. The workability, mechanical, and durability properties were studied, and the changes in the interfacial transition zone (ITZ) of concrete before and after sulfate attack and freeze–thaw cycles were analyzed based on the SEM test. The life prediction of NCCG coarse-aggregate high-performance concrete was carried out based on the grey system GM(1,1) prediction model. The results show that the NCCG coarse-aggregate contents have the greatest effect on compressive strength, sulfate resistance, and frost resistance. The W/B ratio has the greatest effect on the anti-carbonization properties. Fly ash–slag mixing can obtain better durability. Considering the effect on the design service life of high-performance concrete, NCCG coarse aggregate is used to prepare high-performance concrete in North China, and the recommended content is 60%; in the Northwest and Northeast regions, the recommended content is 45%. This study provides a basis for the preparation of high-performance concrete with NCCG coarse aggregate.

## 1. Introduction

Green, low-carbon, and sustainable development has become a global strategy. The coarse aggregate in concrete mostly uses ordinary crushed stone, and with the over-exploitation of natural resources, natural crushed stone aggregates are increasingly being depleted, and their price is rising. The exploitation of a large number of mineral resources has a great impact on the natural environment [1]. Coal gangue is the solid waste discharged in the process of coal mining and washing, accounting for 10~25% of coal mining [2,3]. Large quantities of coal gangue accumulations have an important impact on groundwater, soil, and other environments. Non-spontaneous combustion coal gangue (NCCG) is a newly mined, non-spontaneous combustion, black-gray rock. The most practical and economical method is to use it as concrete aggregate. NCCG as aggregate is an important way to realize bulk utilization of coal gangue and reduce the environmental damage caused by spontaneous combustion of coal gangue accumulation. At the same time, it also reduces the extraction of natural sand and crushed stone aggregate, which is conducive to protecting the ecological environment.

There has been extensive research on the mechanics, shrinkage characteristics, and durability of spontaneous combustion coal gangue as coarse- and fine-aggregate concrete [4,5], while there has been less research on NCCG. The comprehensive utilization of coal gangue has become a popular topic in the industry. At present, coal gangue is mainly used for the construction of roads, railways, sintered bricks, and concrete components [6,7]. Due to the layered characteristics of coal gangue aggregate [3], the content of coal gangue aggregate has the greatest effect on the compressive strength of concrete. Liu et al. [8] used 100% coal gangue to prepare concrete, and the elastic modulus was reduced by 57%. It has also been found that the 28 d elastic modulus of concrete mixed with 100% spontaneous combustion coal gangue decreases by 32% [9]. Zhao et al. [10] found that the original coal gangue has a negative impact on the mechanical properties and durability of concrete, and the higher the content of the original coal gangue, the greater the degradation in the strength, elastic modulus, wear resistance, impermeability, and frost resistance of the concrete. Fly ash has a dual improvement effect on the interfacial transition zone (ITZ) performance and mortar pore structure of coal gangue concrete. Due to the rougher surface of coal gangue compared to crushed stone, the microspheres of fly ash are easily enriched [11]. When the coarse and fine aggregates of concrete are all coal gangue, the compressive strength at 28 d is 52.24 MPa and 44.80 MPa, respectively, when the W/B ratios are 0.4 and 0.5, which meet the strength requirements of C30~C50 concrete [12]. Qiu et al. [13] found that with the increase in the freeze–thaw cycles, the overall change trend of relative mass, compressive strength, and dynamic elastic modulus of coal gangue aggregate concrete decreased gradually, and the above indexes declined rapidly with the increase in coal gangue content.

Durability is an important index used to evaluate the environmental effects of concrete. Ma et al. [2] prepared concrete with original aggregate and 700 °C calcined coal gangue as coarse aggregate. The study found that calcined coal gangue aggregate showed greater advantages in sulfate attack resistance, and the concrete with calcined coal gangue coarse aggregate showed better frost resistance in the early stage. It is recommended that the content of coal gangue coarse aggregate is 30~50%. The freeze–thaw damage value of coal gangue concrete has a linear positive correlation with the freeze–thaw cycles, and the damage value increases rapidly with the increase in coal gangue content [13].

In summary, a large number of researchers have demonstrated that using coal gangue as coarse aggregate is feasible, but a large content of coal gangue has a negative effect on the mechanical and durability of concrete. Few scholars have paid attention to the study of “strong wrapped weak” concrete. Using high-performance mortars to wrap NCCG coarse aggregate to prepare NCCG coarse-aggregate high-performance concrete will greatly improve the durability of concrete. In this study, NCCG was used as a coarse aggregate to prepare high-performance concrete. The research variables include W/B ratios (0.20, 0.22, 0.25, and 0.28), NCCG coarse-aggregate replacement rates (0 vt%, 30 vt%, 45 vt%, and 60 vt%), fly ash–slag mass ratios (replacement rate 30%, fly ash and slag mass ratio 0:3, 1:2, 1:1, 2:1, and 3:0), and silica fume coating (NCCG coarse aggregate coated with silica fume pastes). The workability (slump and expansion), mechanical properties (compressive strength, splitting tensile strength, and axial compressive strength), and durability (frost resistance, sulfate resistance, and carbonation resistance) of concrete were tested. Based on scanning electron microscopy (SEM) testing, the impact mechanism of NCCG coarse aggregate on the ITZ and the durability performance of concrete was analyzed. Finally, based on the grey system GM(1,1) prediction model, the life prediction of NCCG coarse-aggregate high-performance concrete was carried out.

## 2. Materials and Experimental Methods

### 2.1. Raw Materials

**Figure 1:**
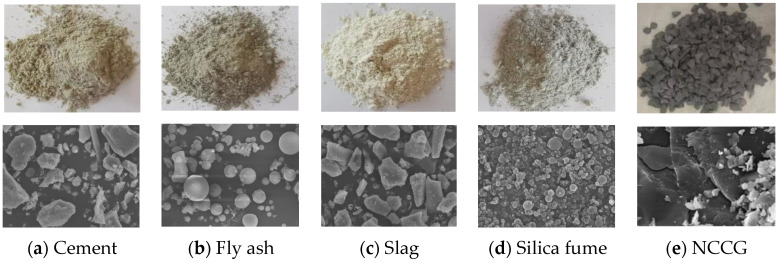
Raw materials and their SEM photos (Magnification factor 2000×).

The cementitious material for this test was P.O 52.5 Portland cement, and the basic indicators are shown in Table 1. The supplementary cementitious materials were Class F fly ash, S95 slag, and silica fume. The chemical composition of the raw materials was obtained by X-ray fluorescence spectrometry, as shown in Table 2. The high-range water reducer (HRWR) selects the BASF polycarboxylic acid high-range water reducer, and the water-reducing effect is about 35~40%. The river sand with a fineness modulus of 2.70 was selected as the fine aggregate. The NCCG and ordinary crushed stone were selected as the coarse aggregate. The main physical performance indexes of the two coarse aggregates were tested, as shown in Table 3. The crushing value and needle and flake content of NCCG are significantly higher than those of ordinary crushed stone, and its apparent density and bulk density are slightly lower than those of ordinary crushed stone, while its mud content and water absorption are significantly higher than those of ordinary crushed stone. The main reason for this is that NCCG has a layered structure, which leads to more needles and a high crushing value after crushing, and more internal pores. Figure 1 shows the raw materials and their SEM photos. Chemically pure isopropanol was used to terminate the hydration of concrete.

### 2.2. Experimental Procedures

In this study, the W/B ratios (0.20, 0.22, 025, and 0.28), NCCG coarse-aggregate replacement rates (0 vt%, 30 vt%, 45 vt% and 60 vt%), fly ash–slag mass rates (0:3, 1:2, 1:1, 2:1 and 3:0, and the total content is 30%), and silica fume coating were considered. NCCG has a high water absorption rate [14], so when preparing concrete, the coarse aggregate was pre-wetted to reduce the intake of mixing water. The pre-wetted water consumption was not included in the test water–binder ratio. Two pretreatment methods were used: pre-wetting treatment and silica fume paste coating treatment. The specific mix proportions are shown in Table 4.

**Figure 2:**
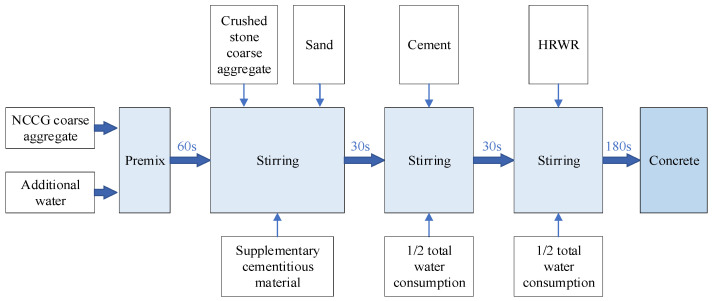
Preparation technology of NCCG coarse-aggregate high-performance concrete.

The preparation process of NCCG coarse-aggregate high-performance concrete is shown in Figure 2. The coal gangue aggregate and additional water were mixed in a mixer for 60 s and stood for 5 min. Then, ordinary crushed stone, sand, and supplementary cementitious material were added and the mixture was stirred for 30 s; cement and 1/2 water consumption were added and the mixture was stirred for 30 s; and, finally, HRWR and 1/2 water consumption were added and the mixture was stirred for 180 s. Firstly, the slump and expansion of fresh concrete were tested, and then the fresh concrete was poured into the molds of 100 mm × 100 mm × 100 mm, 100 mm × 100 mm × 300 mm, and 100 mm × 100 mm × 400 mm. These were covered with plastic wrap and moved to a standard curing room (temperature = 20 ± 2 °C, relative humidity = 95 ± 1%) for 1 day before demolding. Then, samples were moved to the standard curing room for curing until the testing age.

### 2.3. Experimental Setups

The test was divided into three parts: workability, mechanical properties, and durability. The number of test blocks required for mechanical and durability tests is shown in Table 5.

#### 2.3.1. Workability Tests

The workability test refers to the standard GB/T 50080-2016 [15], and the detailed test steps are the same as those in reference [16].

#### 2.3.2. Mechanical Property Tests

**Figure 3:**
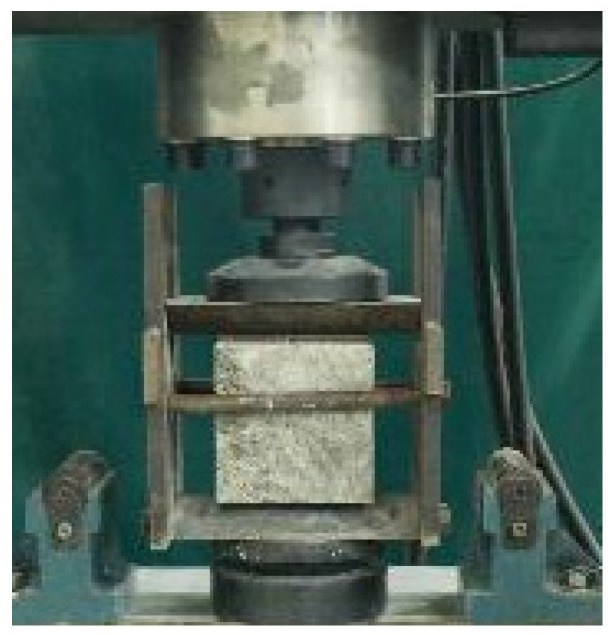
Splitting tensile strength test device.

The sample size for compressive strength and splitting tensile strength testing is 100 mm × 100 mm × 100 mm, and the axial compressive strength is 100 mm × 100 mm × 300 mm. The WAW-1000 micro-controlled electro-hydraulic servo universal testing machine was used to test the samples. Three samples were tested in each group, and the average value of the three samples was taken as the final strength. The splitting tensile strength testing device is shown in Figure 3.

#### 2.3.3. Durability Tests

**(1) Freeze–thaw cycle tests**: According to the GB/T50082-2009 standard [17], a standard rapid freeze–thaw cycle test chamber (temperature −18 °C~5 °C) was used. The number of freeze–thaw cycles was set as 0, 25, 50, 75, 100, 125, and 150, and the measured parameters were compressive strength, mass, relative dynamic elastic modulus, and ultrasonic wave velocity. The mass and relative dynamic elastic modulus were tested using a 100 mm × 100 mm × 400 mm prism test block, and the ultrasonic wave velocity and compressive strength were tested using a 100 mm × 100 mm × 100 mm cube test block.

**(2) Sulfate resistance tests**: According to the GB/T50082-2009 standard, 100 mm × 100 mm × 100 mm samples were immersed in a 5% sodium sulfate solution for a long time, and the solution was changed every 60 days. The sulfate attack age was set at 0 d, 60 d, 120 d, 180 d, and 240 d, and the measured parameters were compressive strength, ultrasonic wave velocity, and mass.

**(3) Carbonization resistance tests**: According to the GB/T50082-2009 standard, the sample size was 100 mm × 100 mm × 400 mm. The two ends of the sample with standard curing for 28 days were sealed with paraffin and placed in a carbonization box. The carbon dioxide concentration was (20 ± 1%), the humidity was (70 ± 5%), and the temperature was (20 ± 2 °C). The carbonization cycle was set as 3 d, 7 d, 14 d, 28 d, 60 d, 100 d, and 200 d; a cutting machine was used to break the section and spray alcohol phenolphthalein was used to obtain the carbonization depth.

#### 2.3.4. Microscopic Tests

**SEM tests**: A concrete sample of about 1 cm was soaked in isopropanol for 48 h and then dried in a low-temperature vacuum drying oven for 48 h. The Hitachi Regulus8100 SEM was used, the test voltage was 20 kV, and the magnification was 500 times and 1000 times. Spraying gold was required before the test.

## 3. Results and Discussions

### 3.1. Workability Analysis

The workability index is an important indicator for evaluating construction performance. Through the mixing process, it was observed that the prepared concrete had good water retention and cohesiveness. Table 6 shows the slump and expansion results of NCCG coarse-aggregate high-performance concrete. Samples A–D were used to test W/B ratio factors, and showed that slump and expansion increased with the increase in W/B ratios. When the W/B ratios increased from 0.20 to 0.28, the slump and expansion increased by 33.33% and 28.76%, respectively. Samples C and E-G were used to test NCCG coarse-aggregate content factors, and showed that the slump and expansion of concrete decreased with the increase in NCCG coarse-aggregate content. Compared with the replacement rate of 0%, the slump with NCCG coarse-aggregate replacement rates of 30%, 45%, and 60% decreased by 3.85%, 9.62%, and 17.31%, respectively; the expansion decreased by 0.64%, 15.90%, and 20.35%, respectively. Mainly because the coal gangue has a layered structure, the contact area between NCCG coarse aggregate and mortar is larger, the bonding performance is stronger, and the slump is reduced. Furthermore, the water absorption of NCCG coarse aggregate will also reduce the slump and expansion of concrete.

The mass ratios of fly ash–slag also affect the workability of concrete. When fly ash content was 30%, the slump and expansion were 270 mm and 638 mm, respectively; when slag content was 30%, the slump and expansion were 230 mm and 576 mm, respectively. Fly ash has a significant improvement effect on the workability of concrete, which is mainly related to the “ball effect” of fly ash particles [18]. Samples C, K, and L were used to test the factors of silica fume coating, and the slump and expansion of concrete were slightly reduced by silica fume coating. The main reason for this is that silica fume has strong water absorption, and some small particles dissolve rapidly after contact with water. The gel, which is rich in SiO_2_ and poor in Ca in the solution, forms an attachment layer on the surface of silica fume particles [19], which increases the viscosity of concrete and reduces the workability of concrete.

### 3.2. Compressive Strength Analysis

**Figure 4:**
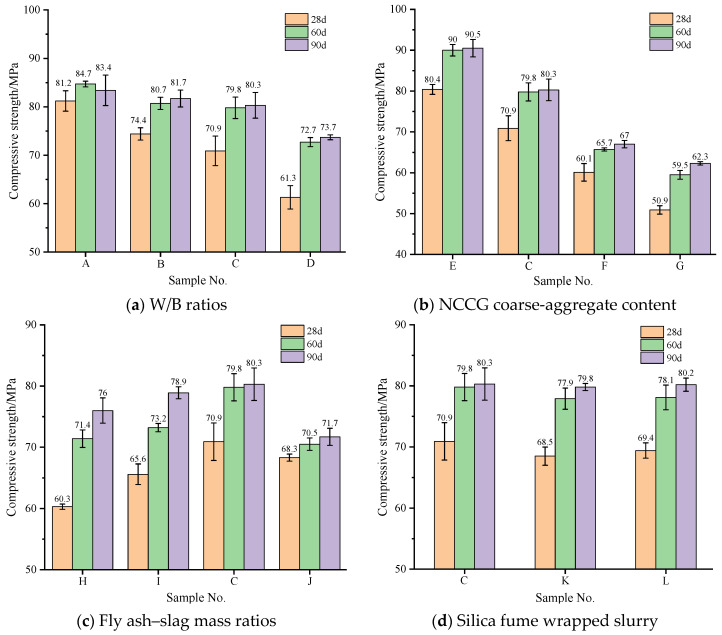
The compressive strength of NCCG coarse-aggregate high-performance concrete.

Figure 4 shows the compressive strength of NCCG coarse-aggregate high-performance concrete, and Figure 4a shows the W/B ratio factor. With the increase in the W/B ratios, the compressive strength of concrete decreased; in particular, the 28 d compressive strength is more significantly affected by the W/B ratios. Figure 4b shows the NCCG coarse-aggregate content factor. As the NCCG coarse-aggregate content increased, the compressive strength continuously decreased. The 28 d compressive strength of NCCG coarse-aggregate high-performance concrete with 0%, 30%, 45%, and 60% content was 80.4 MPa, 70.9 MPa, 60.1 MPa, and 50.9 MPa, respectively. It is obvious that the compressive strength decreases significantly with the increase in NCCG coarse-aggregate content (the compressive strength decreased by 11.82%, 25.25%, and 36.69% when the content was 30%, 45%, and 60%, respectively). This is mainly because the NCCG coarse aggregate itself has a high crushing value and lower strength. Figure 4c shows the fly ash–slag mass ratio factor. When more fly ash was added, the initial compressive strength was lower, and the later strength increased significantly with the increase in curing age. When the slag content was large, the initial strength was large, and the increase rate was slow in the later period. The compressive strength at 90 d is still higher than that of fly ash. Figure 4d shows the silica fume coating factor, which reduces the compressive strength of concrete. With the reduction in the water-silica fume ratio of silica fume coating, the compressive strength decreased. This is due to silica fume being adsorbed on the surface of NCCG coarse aggregate; although this can make the surface of NCCG coarse aggregate denser, it hinders the combination of other cementitious materials with NCCG and reduces the interface bonding force, resulting in a decrease in compressive strength.

### 3.3. Splitting Tensile Strength Analysis

**Figure 5:**
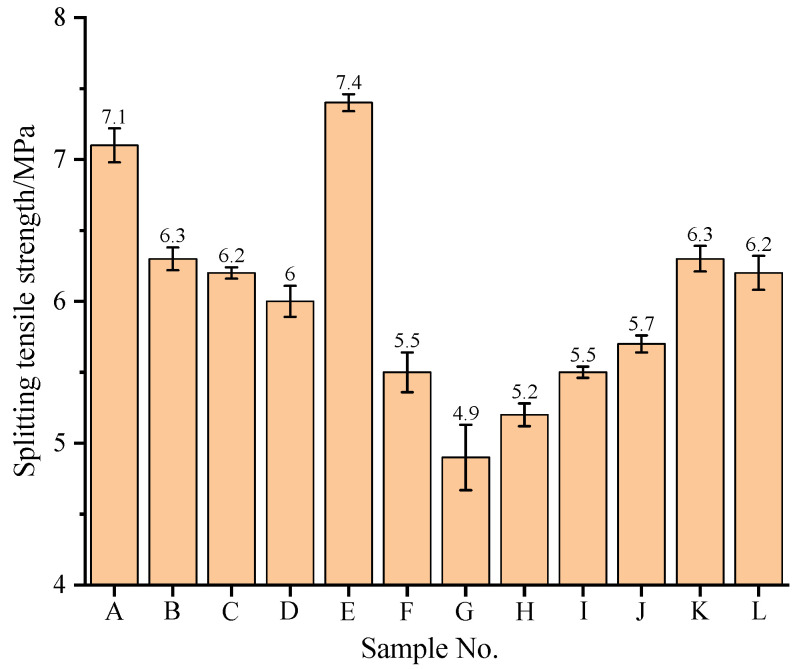
The splitting tensile strength of NCCG coarse-aggregate high-performance concrete.

The splitting tensile strength can be used to evaluate the cracking resistance of concrete. During the test, with the continuous increase in the load, multiple vertical cracks first appeared at the contact between the two ends of the test block and the pad block, and then continued to spread from the two ends of the test block to the middle. When the crack ran through the whole test block, there was a sound, the pressure gauge value fell off, and the test block was crushed. Due to the high crushing value and needle-like content of NCCG itself, it was completely chopped under the splitting action, while ordinary crushed stones are rarely chopped (see Appendix A). Figure 5 shows the splitting tensile strength of NCCG coarse-aggregate high-performance concrete. With the increase in the W/B ratio, the splitting tensile strength decreases. The splitting tensile strength of NCCG coarse-aggregate high-performance concrete with a content of 0%, 30%, 45%, and 60% was 7.4 MPa, 6.2 MPa, 5.5 MPa, and 4.9 MPa, respectively. The splitting tensile strength of concrete with NCCG coarse-aggregate content of 30%, 45%, and 60% decreased by 16.22%, 25.68%, and 33.78%, respectively. The effect of NCCG coarse-aggregate content on the splitting tensile strength of concrete was the most significant. The splitting tensile strength increased first and then decreased with the decrease in the fly ash replacement rate and the increase in the slag replacement rate. When the fly ash–slag mass ratio was 1:2, the splitting tensile strength was the largest. The splitting tensile strength of concrete was slightly improved by silica fume coating, and the smaller the water–silica fume ratio of the silica fume coating, the more the splitting tensile strength increased, mainly due to the smaller particle size of silica fume, which fills the micro-cracks and pores of ITZ.

### 3.4. Axial Compressive Strength Analysis

**Figure 6:**
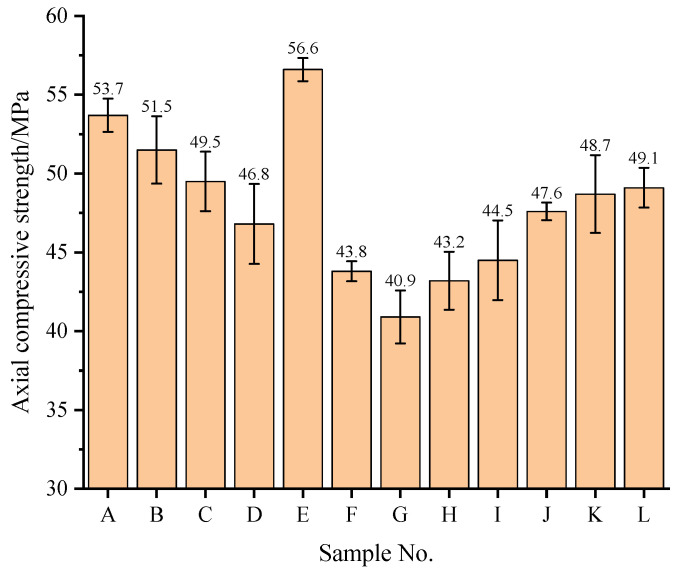
The axial compressive strength of NCCG coarse-aggregate high-performance concrete.

Figure 6 shows the axial compressive strength of NCCG coarse-aggregate high-performance concrete. The axial compressive strength exhibits the same variation as the compressive strength and splitting tensile strength. When the W/B ratio increased from 0.20 to 0.28, the axial compressive strength decreased by 12.85%. When the NCCG coarse-aggregate content increased from 0% to 60%, the axial compressive strength decreased by 27.74%. The factors affecting the axial compressive strength are ranked as NCCG coarse-aggregate content > W/B ratio > fly ash–slag mass ratio > silica fume coating.

### 3.5. Freeze–Thaw Cycle Results Analysis

**Figure 7:**
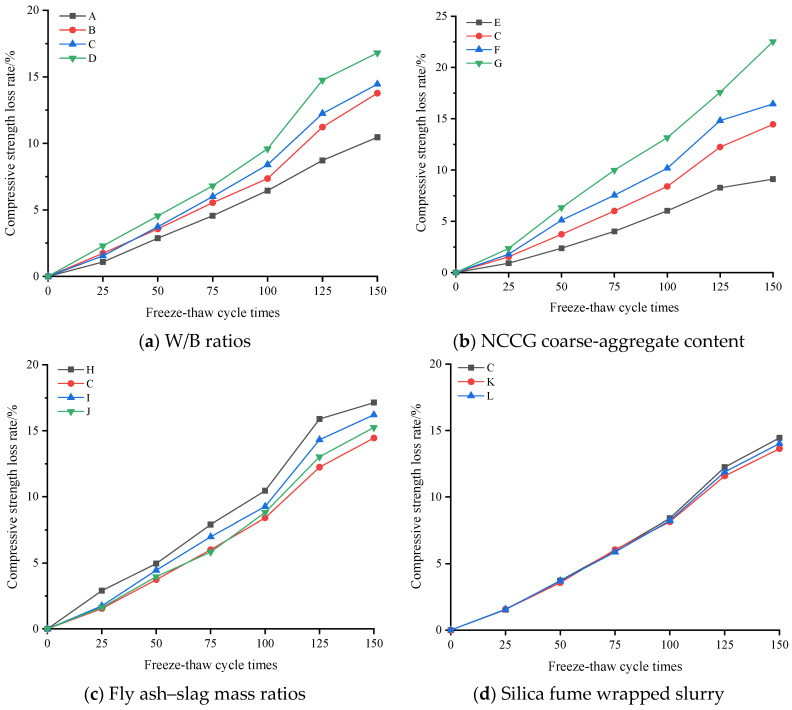
The compressive strength loss rate of NCCG coarse-aggregate high-performance concrete after free have cycles.

**Figure 8:**
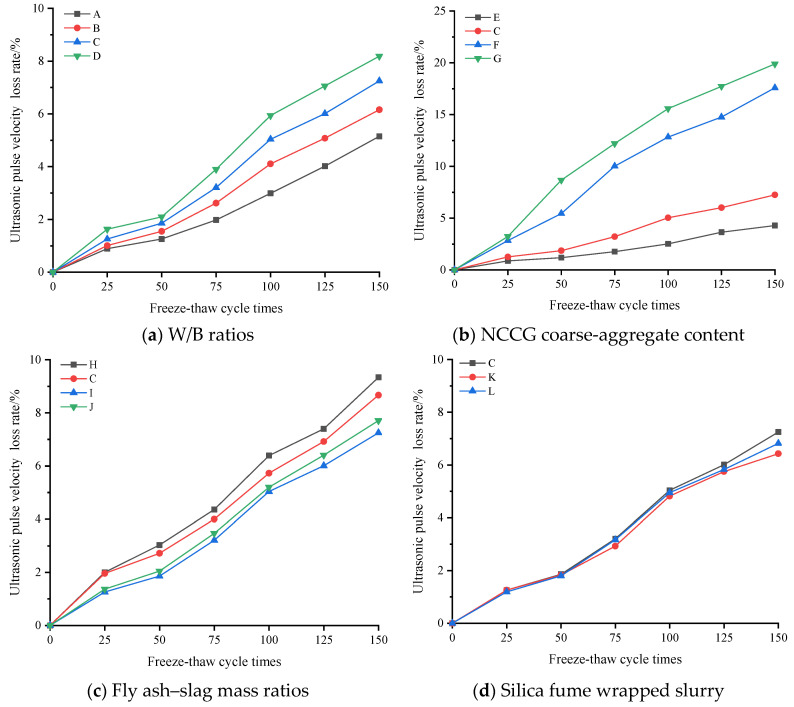
The ultrasonic wave velocity loss rate of NCCG coarse-aggregate high-performance concrete after free have cycles.

Due to the layered structure and large water absorption of coal gangue, relevant studies have found that the frost resistance of coal gangue coarse-aggregate concrete is poor [13]. Figure 7 shows the compressive strength loss rate of NCCG coarse-aggregate high-performance concrete after the freeze–thaw cycles. The compressive strength of all samples decreased with the increase in the number of freeze–thaw cycles. Figure 7a shows the W/B ratio factor. As the W/B ratio increased, the compressive strength loss rate increased. Figure 7b is the NCCG coarse-aggregate content factor. After 150 freeze–thaw cycles, the compressive strength loss rates of concrete with 0%, 30%, 45%, and 60% NCCG coarse-aggregate content were 9.11%, 14.45%, 16.44%, and 22.52%, respectively, and the compressive strength loss rates increased significantly. Figure 7c shows the fly ash–slag mass ratio factor. The concrete sample with only fly ash had the highest compressive strength loss rate (17.13%), and the compressive strength loss rate slightly improved after adding slag. Figure 7d shows the silica fume coating factor. Compared with non-silica-fume-coated concrete, the compressive strength loss rate of concrete with a water silica fume ratio of 0.80 and 0.55 reduced by 0.43% and 0.81%, respectively. Figure 8 shows the ultrasonic wave velocity loss rate of NCCG coarse-aggregate high-performance concrete after freeze–thaw cycles, showing a similar variation to that of the compressive strength loss rate. The NCCG coarse-aggregate content was the biggest influencing factor. After 150 freeze–thaw cycles, the ultrasonic wave velocity loss rates of NCCG coarse-aggregate content of 0%, 30%, 45%, and 60% were 4.29%, 7.25%, 17.58%, and 19.89%, respectively.

**Figure 9:**
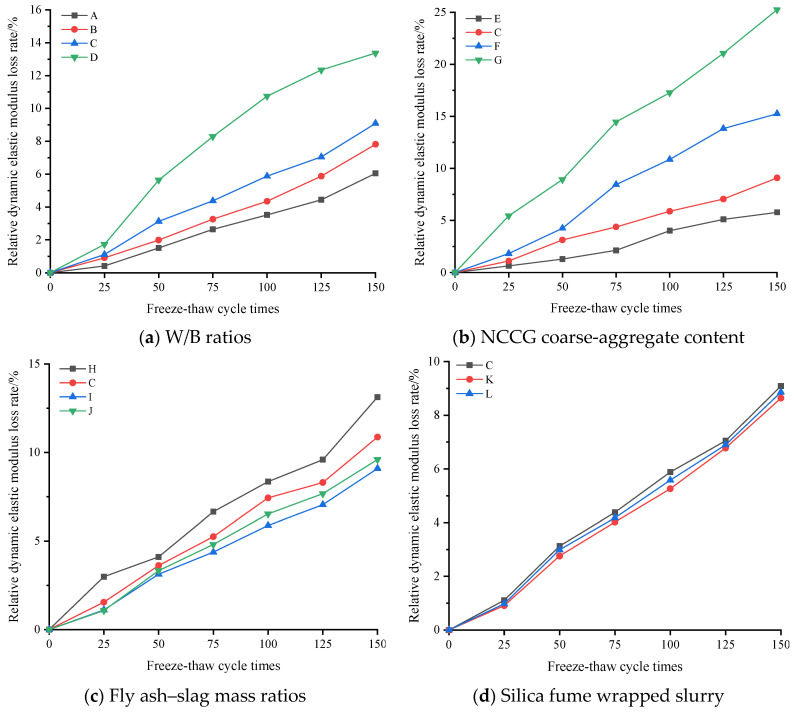
The relative dynamic elastic modulus loss rate of NCCG coarse-aggregate high-performance concrete after freeze–thaw cycles.

**Figure 10:**
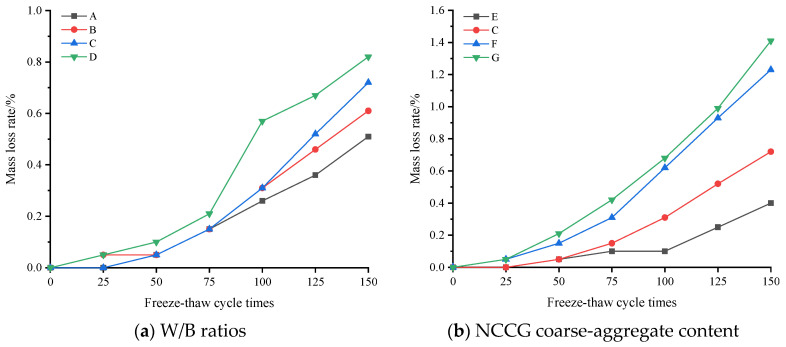

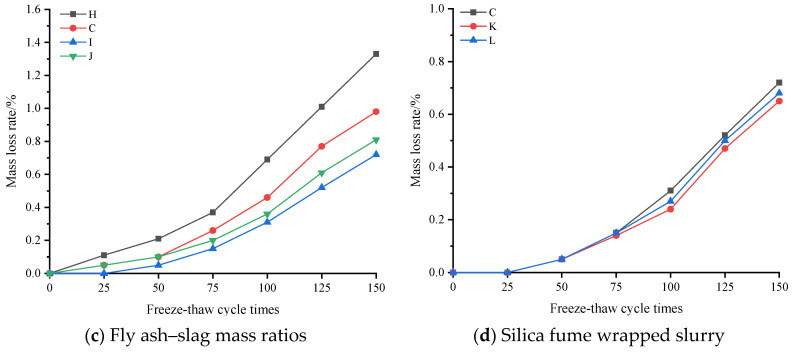
The mass loss rate of NCCG coarse-aggregate high-performance concrete after freeze–thaw cycles.

Figure 9 shows the relative dynamic elastic modulus loss rate of NCCG coarse-aggregate high-performance concrete after freeze–thaw cycles. Figure 9a shows the W/B ratio factor. The larger the W/B ratio, the more the relative dynamic elastic modulus loss, especially when the W/B ratio was 0.28, and the relative dynamic elastic modulus loss rate increased significantly. Figure 9b shows the NCCG coarse-aggregate content factor. The relative dynamic elastic modulus loss rate of concrete with 0%, 30%, 45%, and 60% NCCG coarse-aggregate content was 5.78%, 9.09%, 15.25%, and 25.25%, respectively. When the NCCG coarse-aggregate content exceeded 30%, the relative dynamic elastic modulus loss rate increased rapidly. Figure 9c shows the fly ash–slag mass ratio factor. When fly ash was added alone, the relative dynamic elastic modulus decreased the most, reaching 13.13%. When 30% slag was added alone, the relative dynamic elastic modulus decreased by 9.6%. When 10% fly ash and 20% slag were added, the decrease was the least, reaching 9.09%. This indicates that slag can improve the frost resistance of NCCG coarse-aggregate high-performance concrete more than fly ash. Figure 9d shows the silica fume coating factor, and the relative dynamic modulus loss rate of concrete with a water-silica fume ratio of 0.80 and 0.55 decreased by 0.23% and 0.45%, respectively. Figure 10 shows the mass loss rate of NCCG coarse-aggregate high-performance concrete after freeze–thaw cycles, which exhibited the same variation as compressive strength, ultrasonic wave velocity, and relative dynamic elastic modulus. The content of NCCG coarse aggregate had the greatest influence on the mass loss rate, followed by the W/B ratio. 

The larger the W/B ratio, the greater the number of pores in the concrete. The freezing point of the large-aperture pores is higher than that of the small-aperture pores, resulting in larger expansion stress in the small-aperture pores, causing internal cracking and an increase in the pores in the concrete. This will lead to the decrease in the compressive strength, ultrasonic wave velocity, and relative dynamic elastic modulus. The water absorption of NCCG coarse aggregate is high, and the frost-heave pressure leads to a decrease in strength. When the NCCG coarse-aggregate content exceeded 30%, the ultrasonic wave velocity of concrete decreased obviously, which is mainly related to the damage of NCCG. After 150 freeze–thaw cycles, the relative dynamic elastic modulus of the concrete sample with 60% NCCG coarse-aggregate content decreased by 25.25%, and the mass loss rate was 1.41%. The frost resistance of NCCG coarse-aggregate high-performance concrete was significantly higher than that of coal gangue coarse-aggregate ordinary concrete [5,20], showing excellent frost resistance durability.

### 3.6. Sulfate Resistance Analysis

**Figure 11:**
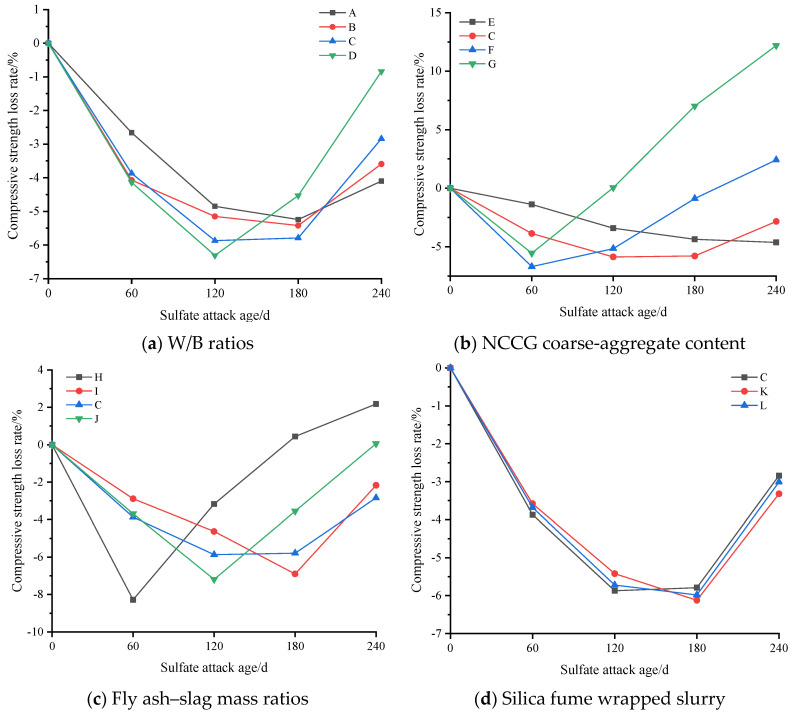
The compressive strength loss rate of NCCG coarse-aggregate high-performance concrete after sulfate attack.

The evaluation indexes of sulfate resistance of NCCG coarse-aggregate concrete are compressive strength loss rate, ultrasonic wave velocity loss rate, and mass loss rate. Figure 11 shows the compressive strength loss rate of NCCG coarse-aggregate high-performance concrete after sulfate attack. Figure 11a shows the W/B ratio factor. With the increase in the W/B ratio, the growth rate of compressive strength at 120 d of sulfate attack increased. This is mainly because with a larger W/B ratio, Na_2_SO_4_ can more easily penetrate into the concrete, resulting in a large amount of ettringite and gypsum, and the increase in compressive strength is more obvious. After 120 d or 180 d of sulfate attack, the compressive strength began to decrease, which was mainly related to the production of expansive ettringite hydration products [21]. Figure 11b shows the NCCG coarse-aggregate content factor, and the four concrete groups show significant differences. When the NCCG coarse-aggregate content was 0%, the compressive strength of concrete after sulfate attack for 240 d still showed an increasing trend. When the NCCG coarse-aggregate content was 30%, the concrete under sulfate attack for 120 d had the highest compressive strength, and then the compressive strength decreased with the increase in attack age. When the NCCG coarse-aggregate content was 45% and 60%, the maximum compressive strength was obtained at 60 d of sulfate attack, and then the compressive strength decreased linearly with the increase in attack age. When NCCG coarse-aggregate content was 60%, the compressive strength loss rate of sulfate attack for 240 d was 12.19%. The main reason for this is that NCCG has a high crushing value, water absorption, and internal porosity, and its internal compactness is low; therefore, sodium sulfate will more easily penetrate into the concrete, and a large amount of ettringite generation causes a faster decline in compressive strength. Figure 11c shows the fly ash–slag mass ratio factor. Single-doped fly ash or slag showed poor resistance to sulfate attack, and the compressive strength decreased by 2.18% and 0.06%, respectively, after 240 d of sulfate attack. The sulfate resistance of composite fly ash–slag concrete was improved, mainly because the specific surface area and surface morphology of fly ash and slag are different, and the mixed use can fill the internal voids of concrete and enhance the compactness of concrete. Figure 11d shows the silica fume coating factor. When NCCG coarse aggregate was treated with silica fume paste coating, the change in sulfate resistance was not significant.

**Figure 12:**
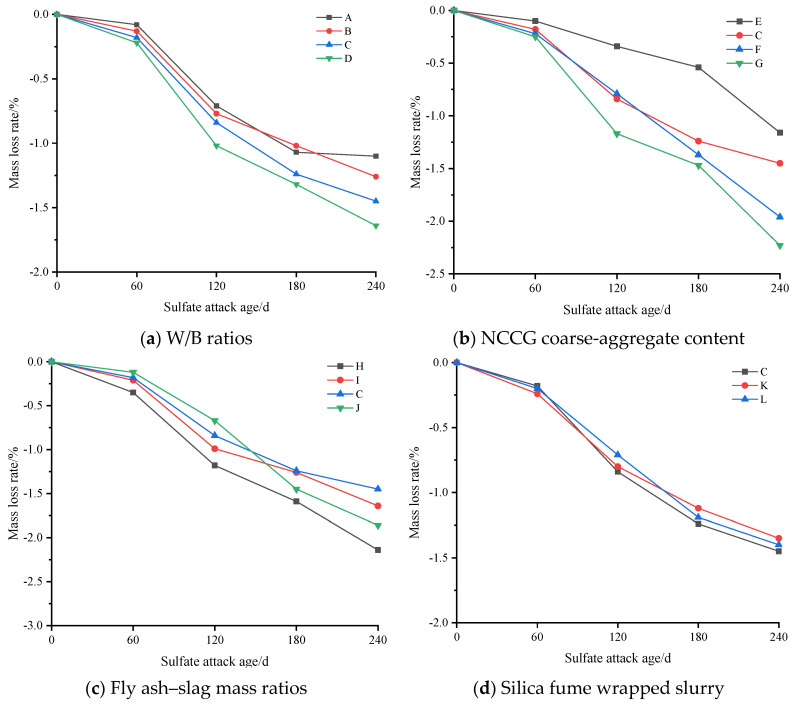
The mass loss rate of NCCG coarse-aggregate high-performance concrete after sulfate attack.

**Figure 13:**
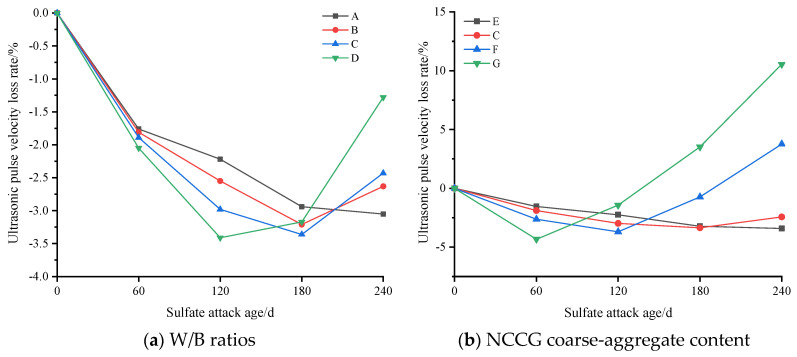

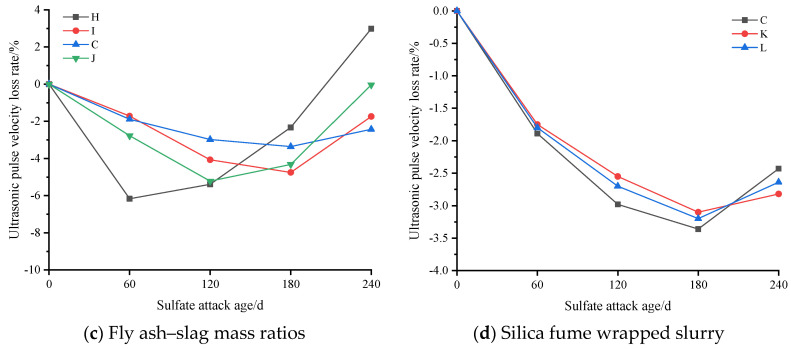
The ultrasonic wave velocity loss rate of NCCG coarse-aggregate high-performance concrete after sulfate attack.

Figure 12 shows the mass loss rate of NCCG coarse-aggregate high-performance concrete after sulfate attack. Comparing the four influencing factors in Figure 12 shows that the NCCG coarse-aggregate content has the greatest effect on the mass loss rate. When the replacement rate was 0%, 30%, 45%, and 60%, the mass of concrete increased by 1.16%, 1.45%, 1.96%, and 2.23% respectively. Due to the use of the same mortar system in the four groups of samples, the increase in mass is related to the adsorption of sulfate by the layered structure of NCCG coarse aggregate and the penetration of the ITZ. With the increase in the W/B ratio, the mass of concrete samples increases more. This is mainly due to Na_2_SO_4_ being more likely to penetrate into the concrete to undergo chemical reactions, generating gypsum and ettringite, resulting in an increase in the mass of the concrete. The mass growth of concrete with two mineral admixtures of fly ash and slag is small, and the mass growth of concrete with only one mineral admixture is large. Ultrasonic wave velocity was used to evaluate the internal homogeneity of concrete. Although concrete is a heterogeneous material, the pores and cracks inside the concrete all affect the ultrasonic wave velocity value. Figure 13 shows the ultrasonic wave velocity loss rate of NCCG coarse-aggregate high-performance concrete after sulfate attack. Overall, the ultrasonic wave velocity and compressive strength of concrete show the same change trend.

### 3.7. Carbonization Resistance Analysis

**Figure 14:**
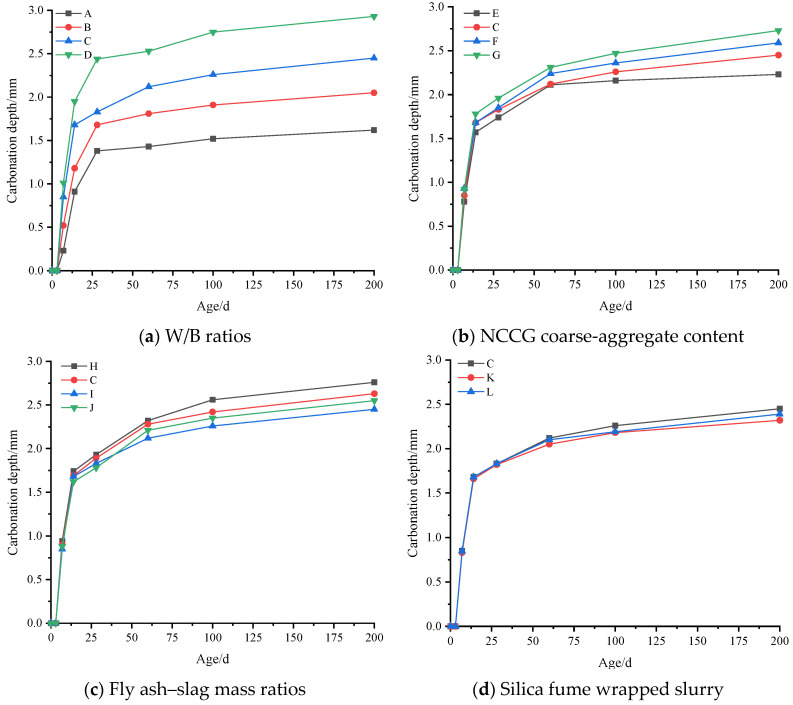
Carbonation depth of NCCG coarse-aggregate high-performance concrete.

Figure 14 shows the carbonation depth of NCCG coarse-aggregate high-performance concrete. Overall, the carbonation depth increases with the increase in carbonation age. In the early stage of carbonation, the carbonation depth increases rapidly, while in the later stage, the carbonation depth increases slowly. Figure 14a shows the W/B ratio factor. As the W/B ratio increases, the carbonation depth increases. When the W/B ratios are 0.20, 0.22, 0.25, and 0.28, the carbonation depths of concrete after 200 d of carbonation are 1.62 mm, 2.05 mm, 2.45 mm, and 2.93 mm, respectively. The W/B ratio has the greatest effect on concrete carbonization, mainly because the larger the W/B ratio, the greater the number of internal pores in NCCG high-performance concrete, thereby providing more diffusion channels for CO_2_. With the increase in carbonization age, the internal compactness of NCCG coarse-aggregate high-performance concrete is high, and CO_2_ reacts with surface Ca(OH)_2_ to generate calcium carbonate, further hindering the invasion of CO_2_. After carbonization of 3 d, due to the dense cement mortar layer on the surface of the concrete, CO_2_ cannot enter, resulting in almost no carbonation depth being measured [22]. Figure 14b shows the NCG coarse-aggregate content factor. With the increase in the NCCG coarse-aggregate content, the carbonation depth of concrete increases. When the NCCG coarse-aggregate content was 60%, the carbonization depth of 200 d concrete was only 2.73 mm, showing excellent carbonization resistance. The NCCG coarse-aggregate content is not the most important factor affecting the carbonization resistance of concrete. In the early stage of carbonization, there is not much difference in the surface cement mortar layer of each group of samples, and CO_2_ enters the interior of the concrete at the same speed. In the later stage of carbonization, due to the layered structure of coal gangue aggregate, there are many micro-cracks and pores, which make it easier for carbon dioxide to diffuse inside the concrete and react with hydration products, resulting in a higher replacement rate of NCCG coarse aggregate and a greater carbonization depth.

Figure 14c shows the fly ash–slag mass ratio factor. The carbonization depth of concrete with fly ash alone is the largest, and the carbonization depth of concrete decreases after the addition of slag. This is mainly related to the Ca(OH)_2_ content in the mortar. The addition of 30% fly ash reduces the Ca(OH)_2_ content in the mortar, which is not conducive to resisting CO_2_ intrusion and leads to an increase in carbonization depth. Figure 14d shows the silica fume coating factor. The NCCG coarse aggregate coated with silica fume can reduce the carbonation depth. The smaller the water–silica fume ratio, the smaller the carbonation depth. When the water–silica fume ratio was 0.55, the carbonization depth of 200 d was 2.32 mm, which was 0.13 mm less than that of uncoated paste. When the water–silica fume ratio was 0.80, the carbonization depth of 200 d was 2.39 mm, which is a reduction of 0.06 mm.

### 3.8. SEM Analysis

**Figure 15:**
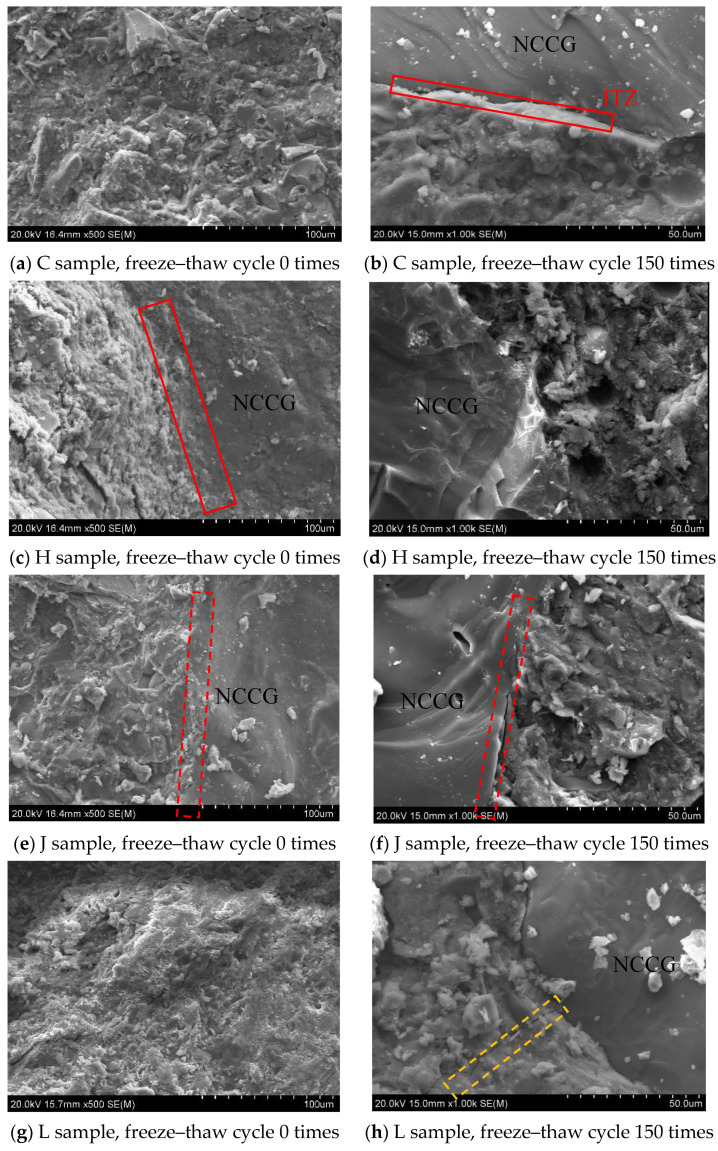
SEM photo of NCCG coarse-aggregate high-performance concrete after freeze–thaw cycles.

**Figure 16:**
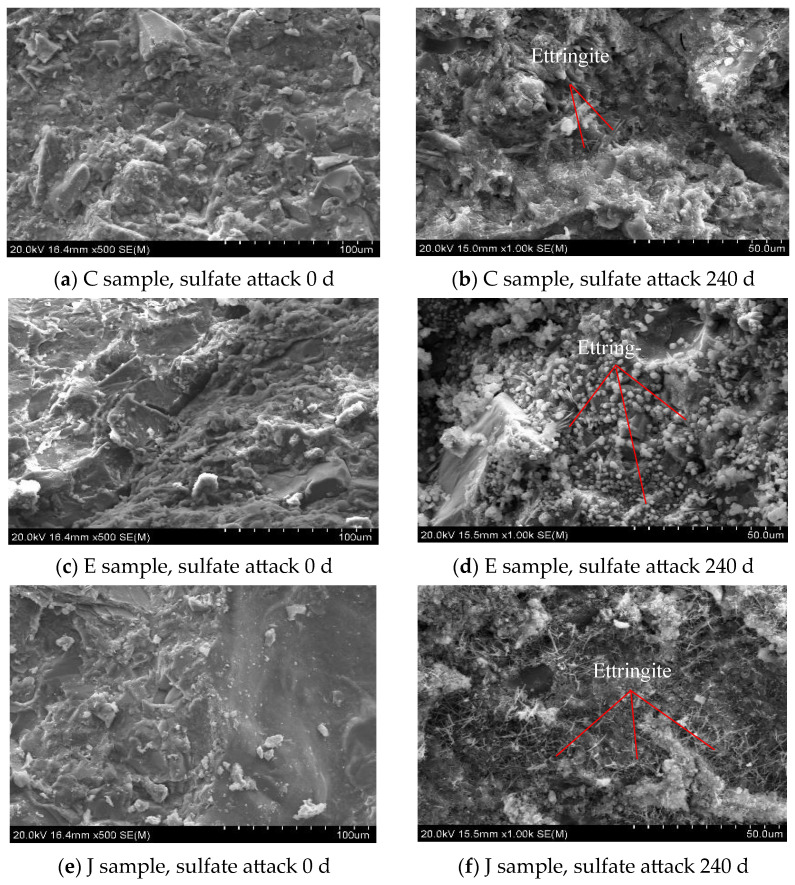
SEM photo of NCCG coarse-aggregate high-performance concrete after sulfate attack.

During the freeze–thaw cycles, the NCCG coarse-aggregate content is the main influencing factor. The concrete samples of Group C, H, J, and L with 0 and 150 freeze–thaw cycles were tested by SEM. Figure 15 shows an SEM photo of NCCG coarse-aggregate high-performance concrete after freeze–thaw cycles. It can be seen that after 150 freeze–thaw cycles, the ITZ of cement mortar and NCCG coarse aggregate have different degrees of damage. The layered structure of NCCG coarse aggregate and the cracks of the ITZ were obviously seen in Group C samples. There is no obvious stratification between NCCG coarse aggregate and mortar after silica-fume-coated paste (L sample) treatment; the crack width is also relatively small and the mortar far away from NCCG coarse aggregate has freeze–thaw cracks, that is, the silica fume coating improves the freeze-resistance of the ITZ. Figure 16 shows the SEM photo of NCCG coarse-aggregate high-performance concrete after sulfate attack. After 240 d of sulfate attack, needle rod and granular ettringite appear in the concrete. It can be clearly seen from Figure 16d,f that the surface of NCCG coarse aggregate is interwoven with a large amount of ettringite.

## 4. Discussion on the Feasibility of Preparing High-Performance Concrete with NCCG Coarse Aggregate

The research on green high-performance concrete mostly focuses on the replacement of cement, focusing on the study of hydration characteristics [23,24,25]. In recent years, many studies have been carried out on the preparation of high-performance concrete by substituting aggregate, including waste glass fine aggregate [26], ceramic aggregate [27], and coral aggregate [28]. Various types of coal gangue, such as calcined coal gangue, spontaneous combustion coal gangue, and non-spontaneous combustion coal gangue, have been proven to be used as coarse aggregate of concrete. However, with the increase in coal gangue content, the mechanical and durability of concrete decrease greatly [3,14,29], and the content of coal gangue should not be too large (≤30%). High-performance concrete takes durability as the main design index. By adding fly ash, slag, and silica fume, the pore structure of the paste is improved and the permeability of the concrete is reduced. Coal gangue is mostly layered rock, and its water absorption rate and crushing index value are higher than those of ordinary crushed stone, which will inevitably have a negative impact on the strength and durability of concrete.

With the increase in NCCG coarse-aggregate content, the compressive strength of concrete decreases approximately linearly. When the NCCG coarse-aggregate content is 60%, the 28 d compressive strength is still greater than 50 MPa, showing excellent compressive strength. Through the durability index test, the NCCG coarse-aggregate content has the greatest effect on frost resistance and sulfate resistance. After 150 freeze–thaw cycles, the mass loss rate of concrete with 60% NCCG coarse-aggregate content is 1.41%, and the relative dynamic elastic modulus loss rate is 25.25%, which is far lower than the requirements of 5% and 40% of the standard specifications, showing excellent frost resistance durability. After 240 d of sulfate attack, the compressive strength of high-performance concrete with 0% and 30% NCCG coarse-aggregate content increased. However, the compressive strength of high-performance concrete with 45% and 60% NCCG coarse-aggregate content decreased by 2.19% and 12.42%, respectively; that is, in a sulfate attack environment, NCCG coarse-aggregate content should not be higher than 45%. NCCG coarse-aggregate content has no significant effect on the anti-carbonization performance. At 20% CO_2_ concentration, the carbonization depth of 200 d is less than 3 mm, showing excellent anti-carbonization performance. In the high-carbonization environment, the NCCG coarse-aggregate content can be greater than 60%.

## 5. Prediction of Frost Resistance Durability Life of NCCG Coarse-Aggregate High-Performance Concrete Based on Grey System GM(1,1)

The service life is related to the performance of the material in a given environment and the deterioration mechanism of the material, which is an inherent property of the material [30]. Most coal mines in China are located in cold regions, such as Northwest, North, and Northeast China, and concrete is mostly subjected to freeze–thaw cycles. Grey system theory is a means to quantitatively predict the future state based on the known information in the evolution process of objective things, and has become an important method for the study of the uncertainty problem [31]. The grey system GM(1,1) refers to a grey model with a first-order variable. With the help of known information or laws to find realistic laws, all the variable sequences called grey can be directly generated by some combination to weaken their randomness and directly show their basic regularity.

### 5.1. Grey System Theory GM(1,1)

(1) Establishment of grey system GM(1,1) prediction model

Set the sequence operator based on the known data as:

X^(0)^ = {X^(0)^(1), X^(0)^(2), …, X^(0)^(n)} (1)

Accumulate X^(0)^ once; the generated sequence of one accumulation is:

X^(1)^ = {X^(1)^(k), k = 1, 2, 3, …, n} (2)

where X^(1)^(k) =$\;\sum_{i=1}^{k}X^{\left(0\right)}(i)\;$= X^(1)^(k−1) + X^(0)^(k), k represents a time series (25 freeze–thaw cycles in this paper).

The first derivative of the time function t and the differential equation in the whitening form of GM(1,1) can be obtained:

(3) $$\frac{dX^{\left(1\right)}}{dt}+aX^{\left(1\right)}=u$$

Equation (3) is the GM(1,1) grey model established in this paper, which is a first-order differential equation. The values of a and u in the equation are calculated from durability test data. Denote the parameter sequence as $\hat{a}$; this can be estimated by the least square method: $\hat{a}={\left[a,u\right]}^{T}$. Suppose that B is a data matrix, Y_n_ is a data column, and the representations of B and Y_n_ are shown in Equations (4) and (5):

(4) $$B=\left[\begin{array}{cc} -\frac{1}{2}(X^{\left(1\right)}\left(1\right)+X^{\left(1\right)}\left(2\right)) & 1\\ \begin{array}{c} -\frac{1}{2}(X^{\left(1\right)}\left(2\right)+X^{\left(1\right)}\left(3\right))\\ \ldots \\ -\frac{1}{2}(X^{\left(1\right)}\left(n-1\right)+X^{\left(1\right)}\left(n\right)) \end{array} & \begin{array}{c} 1\\ \ldots \\ 1 \end{array} \end{array}\right]$$

(5) $${Y_{n}=\left[X^{\left(0\right)}\left(2\right),X^{\left(0\right)}\left(3\right),\cdots ,X^{\left(0\right)}\left(n\right)\right]}^{T}$$

(6) $$The\;calculated\;value\;of\;\hat{a}\;can\;be\;obtained\;based\;on\;B\;and\;Y_{n}:\hat{a}=(B^{T}B{)}^{-1}B^{T}Y_{n}$$

The solution of GM(1,1) whitening equation is calculated as follows:

(7) $${\hat{X}}^{\left(1\right)}(k+1)=\left(X^{\left(0\right)}\left(1\right)-\frac{u}{a}\right)e^{-\mathrm{at}}+\frac{u}{a}$$

(2) Model prediction value restoration

Since the data calculated by the GM(1,1) model represent the one-time cumulative amount of X^(0)^, all the data $\hat{X}$^(1)^(k + 1) obtained by the GM(1,1) model must be reduced to $\hat{X}$^(0)^(k + 1) to be used.

(8) $${\hat{X}}^{\left(1\right)}(k+1)=\sum_{i=1}^{k}{\hat{X}}^{\left(0\right)}(i)={\hat{X}}^{\left(0\right)}\left(k+1\right)+\sum_{i=1}^{k-1}{\hat{X}}^{\left(0\right)}(i)$$

(9) $${{\hat{X}}^{\left(0\right)}(k+1)=\hat{X}}^{\left(1\right)}\left(k+1\right)-\sum_{i=1}^{k-1}{\hat{X}}^{\left(0\right)}(i)$$

(10) $$Due\;to\;{\hat{X}}^{\left(1\right)}(k-1)=\sum_{i=1}^{k-1}{\hat{X}}^{\left(0\right)}(i),i.e.,\;{\hat{X}}^{\left(0\right)}(k+1)={\hat{X}}^{\left(1\right)}\left(k+1\right)-{\hat{X}}^{\left(1\right)}\left(k-1\right)$$

(3) Grey system GM (1,1) prediction model test

GM(1,1) system theory is a prediction calculation method; the accuracy of this prediction model needs to be tested. The commonly used methods include relative error detection, correlation, and mean square error ratio–small error probability calculation [31]. The mean square error ratio–small error probability calculation method is used for testing.

Based on the prediction error ε(*k*) of each group of test blocks, and assuming that X^(0)^ is the original sequence, ${\hat{X}}^{\left(0\right)}$ is the simulation error sequence, and $\varepsilon^{\left(0\right)}$ is the residual sequence, then:

(11) $$The\;mean\;value\;of\;the\;original\;data\;series:\;\bar{X}=\frac{1}{n}\sum_{k=1}^{n}X^{\left(0\right)}(k)$$

(12) $${The\;variance\;of\;the\;original\;data\;series:\;S}_{1}^{2}=\frac{1}{n}\sum_{k=1}^{n}{\left(X^{\left(0\right)}\left(k\right)-\bar{X}\right)}^{2}$$

(13) $$Mean\;residual:\;\bar{\varepsilon }=\frac{1}{n}\sum_{k=1}^{n}\varepsilon (k)$$

(14) $$Residual\;variance:\;S_{2}^{2}=\frac{1}{n}\sum_{k=1}^{n}{\left(\varepsilon (k)-\bar{\varepsilon }\right)}^{2}$$

The mean square error ratio is c = $\;\frac{S_{2}}{S_{1}}$ for a very given c_0_ > 0; when c < c_0_, the model is the mean square error ratio qualified model.

The small error probability value p = P{$\left|\varepsilon (k)-\bar{\varepsilon }\right|<0.6745S_{1}$} for a given p_0_ > 0; when p > p_0_, this model is called a qualified model of the small error probability value. In the grey system GM(1,1) prediction model test, the smaller the c value and the larger the *p* value, the better. According to the c value and *p* value, the prediction model accuracy can be divided into four levels, as shown in Table 7.

### 5.2. Frost Resistance Life of NCCG Coarse-Aggregate High-Performance Concrete

In practical engineering, the frost resistance of concrete is often evaluated in years, rather than times; furthermore, the freeze–thaw cycles vary from place to place, and the same limit times will cause the life of concrete in different regions to fail to meet the predetermined requirements. Therefore, in order to predict the actual life of the concrete using the freeze–thaw test, the measured freeze–thaw index should be transformed into the corresponding years that can be used for the actual calculation. The relationship between the freeze–thaw cycles in the test and nature is expressed as [32]:

(15) $$T=\frac{kn}{N}$$

where *T* is the actual freeze-resistance life of concrete in the natural environment, and n is the number of freeze–thaw cycles when the residual value of the relative dynamic elastic modulus measured by the experiment is 0.6. n is the number of average annual freeze–thaw cycles in each region and *K* is the ratio of the rapid freeze–thaw cycles in the laboratory to the freeze–thaw cycles in nature; the general ratio is 10~15, and 14 was selected for this study. Table 8 shows the annual actual freeze–thaw cycles in Northwest, North, and Northeast China.

The relative dynamic elastic modulus loss rate of less than 0.6 is considered as the freeze–thaw damage standard for concrete. Based on Equations (1)–(14), the accuracy grade of the prediction model of all samples is level one; that is, the grey system theory GM(1,1) can be applied to the prediction of the durability of NCCG coarse-aggregate high-performance concrete. The frost resistance life of NCCG coarse-aggregate high-performance concrete with 12 mix proportions was calculated, as shown in Table 9.

The service life of NCCG high-performance concrete is most affected by the replacement rate of coarse aggregate. The greater the replacement rate, the shorter the service life. In North China, NCCG coarse aggregate is used to prepare high-performance concrete, and the recommended content is 60%. In the Northwest and Northeast regions, the recommended content is 45%. Considering that most of the actual projects are in a humid environment, the freeze–thaw cycle process is carried out under unsaturated water conditions. Therefore, the service life predicted by the grey system GM(1,1) is the lowest service life, and the NCCG coarse-aggregate content can be greatly increased; it is feasible that the NCCG coarse-aggregate content is more than 60%.

## 6. Conclusions

High-performance concrete was prepared with NCCG coarse aggregate, and its workability, mechanical properties, and durability were tested. The service life of concrete was predicted based on the grey system GM(1,1). The chief conclusions of this study can be drawn as follows:

(1) The slump and expansion of concrete decrease with the increase in NCCG coarse-aggregate content. The compressive strength, splitting tensile strength, and axial compressive strength decrease with the increase in NCCG coarse-aggregate content. When the NCCG coarse-aggregate content is 60%, the 28 d compressive strength of concrete is still greater than 50 MPa, showing its excellent mechanical properties.
(2) NCCG coarse-aggregate content has the most significant effect on the freezing resistance and sulfate attack resistance of concrete. When NCCG coarse-aggregate content is 60%, concrete exhibits excellent freezing resistance and sulfate attack resistance. The W/B ratio has the greatest effect on the carbonation resistance of concrete. With the increase in the W/B ratio, the carbonation depth of concrete at 200 d increases. Fly ash–slag mixing can obtain better durability.
(3) After 150 freeze–thaw cycles, the ITZ of cement mortar and NCCG coarse aggregate had different degrees of damage, and the cracks of the ITZ were clearly seen. There is no obvious stratification between NCCG coarse aggregate and mortar after silica fume coating treatment, and the crack width is relatively small. After 240 d of sulfate attack, there are obvious needle-rod and granular ettringite in NCCG coarse-aggregate high-performance concrete.
(4) The replacement rate of NCCG coarse aggregate has the greatest influence on the service life of the concrete. The larger the replacement rate, the shorter the service life of concrete. In North China, NCCG coarse aggregate is used to prepare high-performance concrete, and the recommended content is 60%. In the Northwest and Northeast regions, the recommended content is 45%. In summary, NCCG coarse aggregate can be used to prepare high-performance concrete, and the content of NCCG coarse aggregate can be appropriately increased (>60%) when used in the natural environment.

## Figures and Tables

**Table 1:** Basic indicators of P.O 52.5 Portland cement.

Standard Consistency/%	Loss on Ignition/%	Setting Time/min		Flexural Strength/MPa		Compressive Strength/MPa		Specific Surface Area/(m^2^·kg^−1^)
		Initial	Final	3 d	28 d	3 d	28 d	
28	1.4	115	184	6.2	8.9	33.8	60.2	381

**Table 2:** Main chemical compositions of raw materials/%.

Materials	CaO	MgO	Al_2_O_3_	SiO_2_	Fe_2_O_3_	SO_3_	LOI
OPC	56.77	3.50	5.90	20.86	3.16	2.43	2.06
Fly ash	0.80	0.51	28.10	50.20	2.52	0.29	1.51
Slag	36.29	12.15	13.58	32.21	1.21	0.35	2.31
Silica fume	—	—	—	96.20	—	—	2.60

**Table 3:** Main physical performance index of coarse aggregate.

Physical Performance	NCCG	Ordinary Crushed Stone
Particle size distribution/mm	5~15	5~15
Crushing value/%	18.20	11.20
Apparent density/(kg·m^−3^)	2610	2801
Bulk density/(kg·m^−3^)	1402	1830
Needle and flake content particles/%	11.50	1.50
Mud content/%	6.90	2.30
Water absorption rate/%	5.20	2.70

**Table 4:** Mix proportions of the NCCG coarse-aggregate high-performance concrete/(kg·m^−3^).

Sample No.	Cement	Fly Ash	Slag	Silica Fume	NCCG	Crushed Stone	Sand	Water	HRWR	Additional Water Volume
A	560	80	160	—	271.96	634.58	536.44	160	7.00	7.75
B	560	80	160	—	271.96	634.58	536.44	176	6.36	7.75
C	560	80	160	—	271.96	634.58	536.44	200	5.60	7.75
D	560	80	160	—	271.96	634.58	536.44	224	5.00	7.75
E	560	80	160	—	0	921.57	536.44	200	5.60	0
F	560	80	160	—	404.65	494.57	536.44	200	5.60	11.53
G	560	80	160	—	535.20	356.80	536.44	200	5.60	15.25
H	560	240	0	—	271.96	634.58	536.44	200	5.60	7.75
I	560	160	80	—	271.96	634.58	536.44	200	5.60	7.75
G	560	0	240	—	271.96	634.58	536.44	200	5.60	7.75
K	560	80	160	14.1	271.96	634.58	536.44	200	5.60	7.75
L	560	80	160	9.7	271.96	634.58	536.44	200	5.60	7.75

**Table 5:** The sample number of mechanical and durability tests.

Test	Sample Size	Test Age	Single Sample	Total Quantity
Compressive strength	100 mm × 100 mm × 100 mm	28 d, 60 d and 90 d	9	108
Splitting tensile strength	100 mm × 100 mm × 100 mm	28 d	3	36
Axial compressive strength	100 mm × 100 mm × 300 mm	28 d	3	36
Freeze–thaw cycles	100 mm × 100 mm × 400 mm and 100 mm × 100 mm × 100 mm	0, 25, 50, 75, 100, 125 and 150 times	18 and 18	432
Carbonation resistance	100 mm × 100 mm × 400 mm	0 d, 3 d, 7 d, 14 d, 28 d, 60 d, 100 d and 200 d	1	12
Sulfate attack resistance	100 mm × 100 mm × 100 mm	0 d, 60 d, 120 d, 180 d and 240 d	15	180

**Table 6:** Slump and expansion degree of NCCG coarse-aggregate high-performance concrete.

Sample No.	Slump/mm	Expansion Degree/mm	Sample No.	Slump/mm	Expansion Degree/mm
A	210	518	G	215	501
B	235	575	H	270	628
C	250	625	I	255	622
D	280	667	J	230	547
E	260	629	K	240	601
F	235	529	L	245	594

**Table 7:** Reference table for accuracy inspection grade.

Accuracy Class	Mean-Square Error	Minimum Error
Level 1	c ≤ 0.35	p ≥ 0.95
Level 2	0.35 < c ≤ 0.50	0.80 ≤ p < 0.95
Level 3	0.50 < c ≤ 0.65	0.70 ≤ p < 0.80
Level 4	0.65 < c ≤ 0.80	0.60 ≤ p < 0.70

**Table 8:** The annual average of freeze–thaw cycles in Northern China.

Region	Extreme Minimum Temperature/°C	Annual Mean Temperature Difference/°C	Annual Mean Low Temperature Days/d	Annual Mean of Freeze–Thaw Cycles/N
Northwest	−26.6	52.1	169	118
North	−27.4	53.2	120	84
Northeast	−36.5	66.5	172	120

**Table 9:** NCCG coarse-aggregate high-performance concrete life prediction/year.

Region	Northwest	North	Northeast
A	172	242	169
B	133	188	131
C	116	163	114
D	101	142	99
E	208	292	204
F	44	63	44
G	39	54	38
H	95	133	93
I	104	146	102
J	110	154	108
K	131	183	128
L	122	171	120

## Data Availability

Data are contained within the article.
